# Variation of mitochondrial minichromosome composition in *Hoplopleura* lice (Phthiraptera: Hoplopleuridae) from rats

**DOI:** 10.1186/s13071-020-04381-y

**Published:** 2020-10-06

**Authors:** Yi-Tian Fu, Yu Nie, De-Yong Duan, Guo-Hua Liu

**Affiliations:** 1 Hunan Provincial Key Laboratory of Protein Engineering in Animal Vaccines, College of Veterinary Medicine, Hunan Agricultural University, Changsha, 410128 Hunan China; 2 Hunan Co-Innovation Center of Animal Production Safety, Changsha, 410128 Hunan People’s Republic of China

**Keywords:** Rat louse, Fragmented mt genome, Phylogenetic analyses

## Abstract

**Background:**

The family Hoplopleuridae contains at least 183 species of blood-sucking lice, which widely parasitize both mice and rats. Fragmented mitochondrial (mt) genomes have been reported in two rat lice (*Hoplopleura kitti* and *H. akanezumi*) from this family, but some minichromosomes were unidentified in their mt genomes.

**Methods:**

We sequenced the mt genome of the rat louse *Hoplopleura* sp. with an Illumina platform and compared its mt genome organization with *H. kitti* and *H. akanezumi*.

**Results:**

Fragmented mt genome of the rat louse *Hoplopleura* sp. contains 37 genes which are on 12 circular mt minichromosomes. Each mt minichromosome is 1.8–2.7 kb long and contains 1–5 genes and one large non-coding region. The gene content and arrangement of mt minichromosomes of *Hoplopleura* sp. (*n* = 3) and *H. kitti* (*n* = 3) are different from those in *H. akanezumi* (*n* = 3). Phylogenetic analyses based on the deduced amino acid sequences of the eight protein-coding genes showed that the *Hoplopleura* sp. was more closely related to *H. akanezumi* than to *H. kitti*, and then they formed a monophyletic group.

**Conclusions:**

Comparison among the three rat lice revealed variation in the composition of mt minichromosomes within the genus *Hoplopleura*. *Hoplopleura* sp. is the first species from the family Hoplopleuridae for which a complete fragmented mt genome has been sequenced. The new data provide useful genetic markers for studying the population genetics, molecular systematics and phylogenetics of blood-sucking lice.
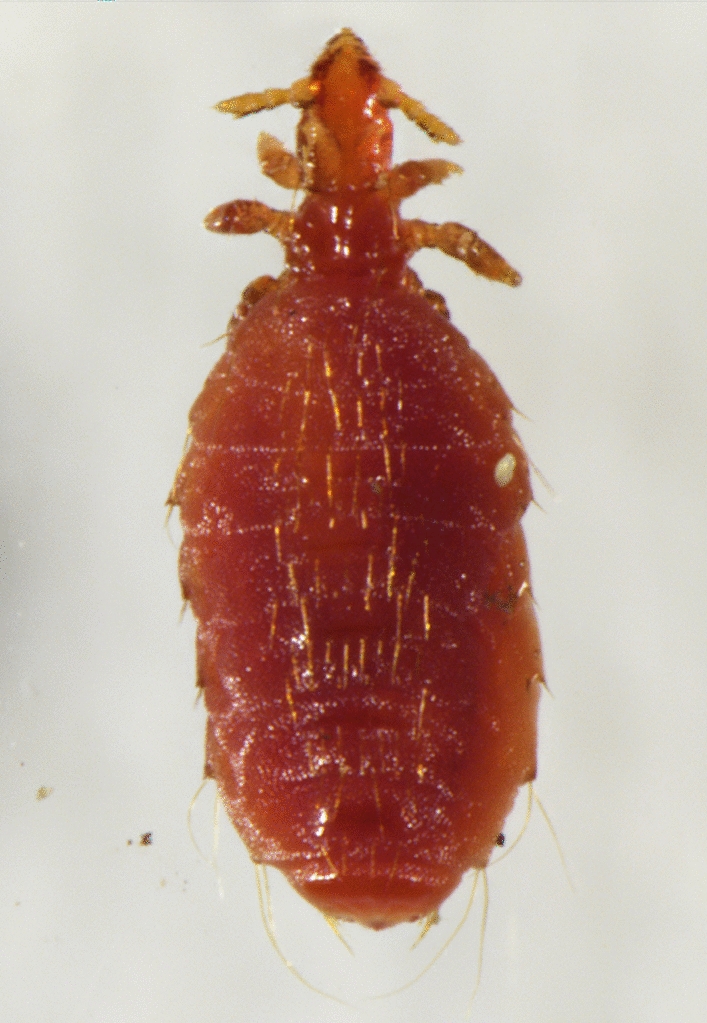

## Background

Blood-sucking lice are known vectors and transmit various disease agents and cause significant vector-borne diseases in humans, domestic and wild mammals [1]. The family Hoplopleuridae contains at least 183 described species of blood-sucking lice currently classified into eight genera [2]. Of the eight genera, *Hoplopleura* Enderlein, 1904 is the most species-rich (165 described species) found on rodents [3]. The *Hoplopleura* spp*.* are common ectoparasites of both mice and rats, causing pruritus, alopecia, dermal irritation and even anemia.

Metazoan mitochondrial (mt) genomes are usually circular DNA molecules (13–20 kb) with 36–37 genes that contain 12–13 protein-coding genes, two rRNA genes and 22 tRNA genes [4]. Some parasitic lice have an unusual, fragmented mt genome organization, but not all species of parasitic lice have been shown to have a fragmented genome. Fragmentation of the mt genome was first found in the human body louse, *Pediculus humanus corporis* (suborder Anoplura) [5]. Since then, 11 other blood-sucking lice, *P. humanus capitis*, *P. schaeffi*, *Pthirus pubis*, *Haematopinus suis*, *H. apri*, *H. asini*, *H. akanezumi*, *H. kitti*, *Polyplax asiatica*, *P. spinulosa* and *Microthoracius praelongiceps* (suborder Anoplura); eight avian feather lice, *Bovicola bovis*, *B. ovis*, *B. caprae*, *Trichodectes canis, Columbicola columbae*, *C. macrourae*, *C. passerinae* 1 and 2 (suborder Ischnocera), and the elephant louse, *Haematomyzus elephantis* (suborder Rhynchophthirina), have been found with fragmented mt genomes [6–15]. While seven feather lice, *Colpocephalum griffoneae*, *Amyrsidea minuta* and *Heterodoxus macropus* (suborder Amblycera), *Ibidoecus bisignatus*, *Campanulotes compar*, *Bothriometopus macrocnemis* and *Falcolipeurus quadripustulatus* (suborder Ischnocera), do not have fragmented mt genomes [15]. To date, the complete mt genomes of 12 blood-sucking lice have been sequenced and deposited in GenBank, but the complete mt genomes have been only reported for two rat lice (*H. kitti* and *H. akanezumi*) from this family Hoplopleuridae [7]. In addition, three genes (*nad*1, *nad*3 and *nad*5) or minichromosomes were unidentified in the mt genomes of two *Hoplopleura* spp. [7]. Interestingly, gene rearrangement has been reported in the fragmented mt genome of two *Hoplopleura* spp. [7]. Therefore, *Hoplopleura* mt genomes may represent one of the most frequently rearranged/fragmented mt genomes within the family Hoplopleuridae.

To understand the composition of mt minichromosomes in species of the same genus, *Hoplopleura*, we sequenced the complete mt genome of the rat louse *Hoplopleura* sp. and compared its mt genome organization with other two *Hoplopleura* species, and re-constructed its phylogenetic relationships within the suborder Anoplura using protein sequences derived from coding genes.

## Methods

### Sample collection and DNA extraction

Adult specimens of *Hoplopleura* sp. were collected from the Edward’s long-tailed rats *Leopoldamys edwardsi* in Chongqing, China. The specific identity of the examined wild rats was determined by PCR-based sequencing of the mitochondrial (mt) *cox*1 gene using an established method [16]. These rat lice were washed five times in physiological saline solution, identified preliminarily to the genus level (as *Hoplopleura* sp.) based on morphological features [2], and stored in 70% (v/v) ethanol at − 20 °C. Whole genomic DNA including nuclear and mt DNA was extracted from 50 single rat lice (25 females and 25 males) using the DNeasy Tissue Kit (Promega, Madison, USA) according to the manufacturer’s recommendations. The identity of these specimens was further confirmed by polymerase chain reaction (PCR) amplification and subsequent sequencing of the mt *cox*1 and *rrn*S genes using primer pairs L6625 (5′-CCG GAT CCT TYT GRT TYT TYG GNC AYC C-3′) and H7005 (5′-CCG GAT CCA CNA CRT ART ANG TRT CRT G-3′), and 12SA (5′-TAC TAT GTT ACG ACT TAT-3′) and 12SB (5′-AAA CTA GGA TTA GAT ACC C-3′), respectively.

### Sequencing and assembling

The purity of the extracted whole genomic DNA was assessed by agarose-gel electrophoresis [17]. The DNA concentration was determined using a Quantus Fluorometer (Invitrogen, Carlsbad, USA). A paired-end genomic DNA library (350 bp inserts) was constructed for high throughput sequencing with Miseq PE300 (Illumina, San Diego, CA, USA) and collected raw reads were exported in the FASTQ format. The raw reads were filtered by removing adaptor reads, redundant reads and ‘N’-rich reads. Finally, 2 Gb clean data (256 bp pair-end reads) was produced for this rat louse. Contigs were de novo assembled from Illumina sequence reads using Geneious 11.1.5 [18] based on *cox*1 and *rrn*S relatively conserved sequences. The assembly parameters were minimum overlap identity 99% and minimum overlap 150 bp. The two ends of the contig overlapped, indicating circular organization of the minichromosome. We observed in previous studies that each mt minichromosome has a distinct coding region but a well-conserved non-coding region [10–13]. The conserved non-coding region sequences were identified between the *cox*1 and *rrn*S minichromosomes and were used as references to align the Illumina sequence dataset. BLAST was used for alignment. We assembled these minichromosomes individually in full length using the same method stated above for *cox*1 and *rrn*S minichromosome assembly.

### Annotation

Sequences were aligned against the mt minichromosome sequences of the rat louse *H. kitti* [7] available using the MAFFT 7.122 software [19] to identify gene boundaries. Protein-coding genes and rRNA genes were identified with BLAST searches of the NCBI database. Amino acid sequences of each protein-coding genes were inferred using MEGA 6.0 [20]. tRNA genes were identified using ARWEN [21] and the program tRNAscan-SE [22] with manual adjustment.

### Verification of mt minichromosomes

**Fig. 1:**
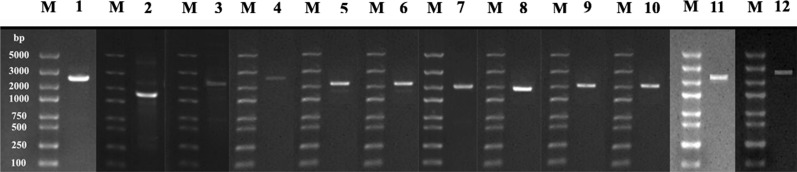
PCR verification of the 12 mt minichromosomes of the rat louse, *Hoplopleura* sp. Lane M: DL2000 DNA marker; Lane 1–12: I-*cox*1, *rrn*S, M-L1-*rrn*L-V, Q-*nad*1-G-*nad*3, D-Y-*cox*2-T, *atp*8-*atp*6-N, *nad*2, C-*nad*6-W-L2, R-*nad*4L-P-*cox*3-A, E-*cyt*b-S1-S2, K -*nad*4 and H-*nad*5-F

The size of each mt minichromosome of *Hoplopleura* sp. were verified by PCR using specific primers (Table 1). The forward primer and reverse primer in each pair were next to each other with a small gap in between (10–50 bp). PCR with these primers amplified each circular minichromosome in full length (Fig. 1). To obtain full-length sequences of the non-coding regions of the minichromosomes, these positive amplicons were also sequenced with high throughput sequencing as described above.

**Table 1:** PCR primers used to amplify and sequence the mitochondrial genome of the rat lice, *Hoplopleura* sp.

Primer	Sequence (5′–3′)	Minichromosome
1F	AGCACTTGTTCTGATTCTTCGGTC	I-*cox*1
1R	TCGTGATACCCCCTGCCAAAACTG	I-*cox*1
2F	CTTTCAAGAGACACAAGGGGTTCA	*rrn*S
2R	TATTTTCCCAGTCCTACAGAGAGC	*rrn*S
3F	TGTCCTTGTCCCGAAAGAGAGTGAT	M-L1-*rrn*L-V
3R	CTATTCCACCCTCCCTGATACAAAA	M-L1-*rrn*L-V
4F	TGAGTAAGGGGGATACATCACGCTA	Q-*nad*1-G-*nad*3
4R	CAGCGAACTCTGCGTATTCCTCCAT	Q-*nad*1-G-*nad*3
5F	TAAGGTTATCGGGCATCAGTGGTA	D-Y-*cox*2-T
5R	AGAGGGGATGGCGAGGACAAAAAG	D-Y-*cox*2-T
6F	CGCCAACTATCAGAACTTTCCAAC	*atp*8-*atp*6-N
6R	TCGTGGATAACAGTCACAAAGATG	*atp*8-*atp*6-N
7F	GCATTTACAGTGCTCAGTCTTCGC	*nad*2
7R	ACAAAGACAAAGGGGGAAACGGGA	*nad*2
8F	TTAGCGGTAAGCGGGACTGAGGTA	C-*nad*6-W-L2
8R	AACTCTATTTCCCCCGTTTCCCAA	C-*nad*6-W-L2
9F	GTTCCTCTCGGTTTTCCATCCCTCA	R-*nad*4L-P-*cox*3-A
9R	TCTATCGCTACCAGAGAGATTGTTA	R-*nad*4L-P-*cox*3-A
10F	GGGAAAACTCCGACAAGGTCACATT	E-*cyt*b-S1-S2
10R	CCTAAGGGATTTGAACTTCCTGTCG	E-*cyt*b-S1-S2
11F	GGTATTGCTAAAGTTTGGAGGTATC	K -*nad*4
11R	CAGCCAAGAGTATTCTCCCCAACAT	K -*nad*4
12F	GGGGATTACCTCCTTCCTTCTCATT	H-*nad*5-F
12R	AAGCAATGAAGAGCAACAAGGACAC	H-*nad*5-F

### Phylogenetic analysis

The phylogenetic relationships among representatives of the blood-sucking lice of suborder Anoplura were assessed based on concatenated amino acid sequences (Table 2), using one elephant louse, *H. elephantis* (GenBank: KF933032-41) as an outgroup [10]. Eight amino acid sequences (except for *nad*1, *nad*2, *nad*3, *nad*4 and *nad*5 because these genes were unidentified in some blood-sucking lice) were aligned individually using MAFFT 7.122 and were then concatenated to form a single dataset; ambiguously aligned regions were excluded using Gblocks 0.91b using default parameters [23]. The MtArt + I + G + F was selected as the most appropriate evolutionary model by ProtTest 2.4 based on the Akaike information criterion (AIC) [24]. Phylogenetic analyses were conducted with maximum likelihood (ML) using PhyML 3.0 with a BioNJ starting tree, and tree topology search was set from the subtree pruning and regrafting (SPR) method [25]. Bootstrap value was calculated using 100 bootstrap replicates. Phylograms were drawn using FigTree v.1.31.

**Table 2:** The blood-sucking lice included in the phylogenetic analyses in this study

Species	Host	GenBank ID	References
*Haematopinus apri*	Wild pig	KC814611-19	[6]
*Haematopinus asini*	Horse	KF939318, KF939322, KF939324, KF939326, KJ43403438	[13]
*Haematopinus suis*	Domestic pig	KC814602-10	[6]
*Hoplopleura akanezumi*	Rat	KJ648922-32	[7]
*Hoplopleura kitti*	Rat	KJ648933-43	[7]
*Microthoradus praelongiceps*	Guanacos	KX090378-KX090389	[11]
*Pediculus humanus corporis*	Human	FJ499473-90	[5]
*Pediculus humanus capitis*	Human	JX080388-407	[12]
*Pediculus schaeffi*	Chimpanzee	KC241882-97, KR706168-69	[9]
*Pthirus pubis*	Human	JQ976018, EU219987-95, HM241895-8	[12]
*Polyplax asiatica*	Rat	KF647751-61	[8]
*Polyplax spinulosa*	Rat	KF647762-72	[8]
*Hoplopleura* sp.	Rat	MT792483-94	Present study

## Results and discussion

### Identity of the rat louse *Hoplopleura* sp.

Two blood-sucking louse species (*H. kitti* and *P. insulsa*) parasitize *L. edwardsi* (https://phthiraptera.info/category/mammal-wilson-reeder/mammals/rodentia/muridae/murinae/leopoldamys/leopoldamys-edwardsi). The *Hoplopleura* sp. has close morphological and morphometric similarities with *H. kitti* recovered from the same host (*L. edwardsi*). The mt *cox*1 and *rrn*S genes of *Hoplopleura* sp. shared 76% and 77.6% identity with previously published sequences of *H. kitti* (GenBank: KJ648943) from *Berylmys bowersi* and *H. akanezumi* (GenBank: KJ648928) from *Apodemus chevrieri* in China, respectively.

### General features of the mt genome of the rat louse *Hoplopleura* sp.

**Fig. 2:**
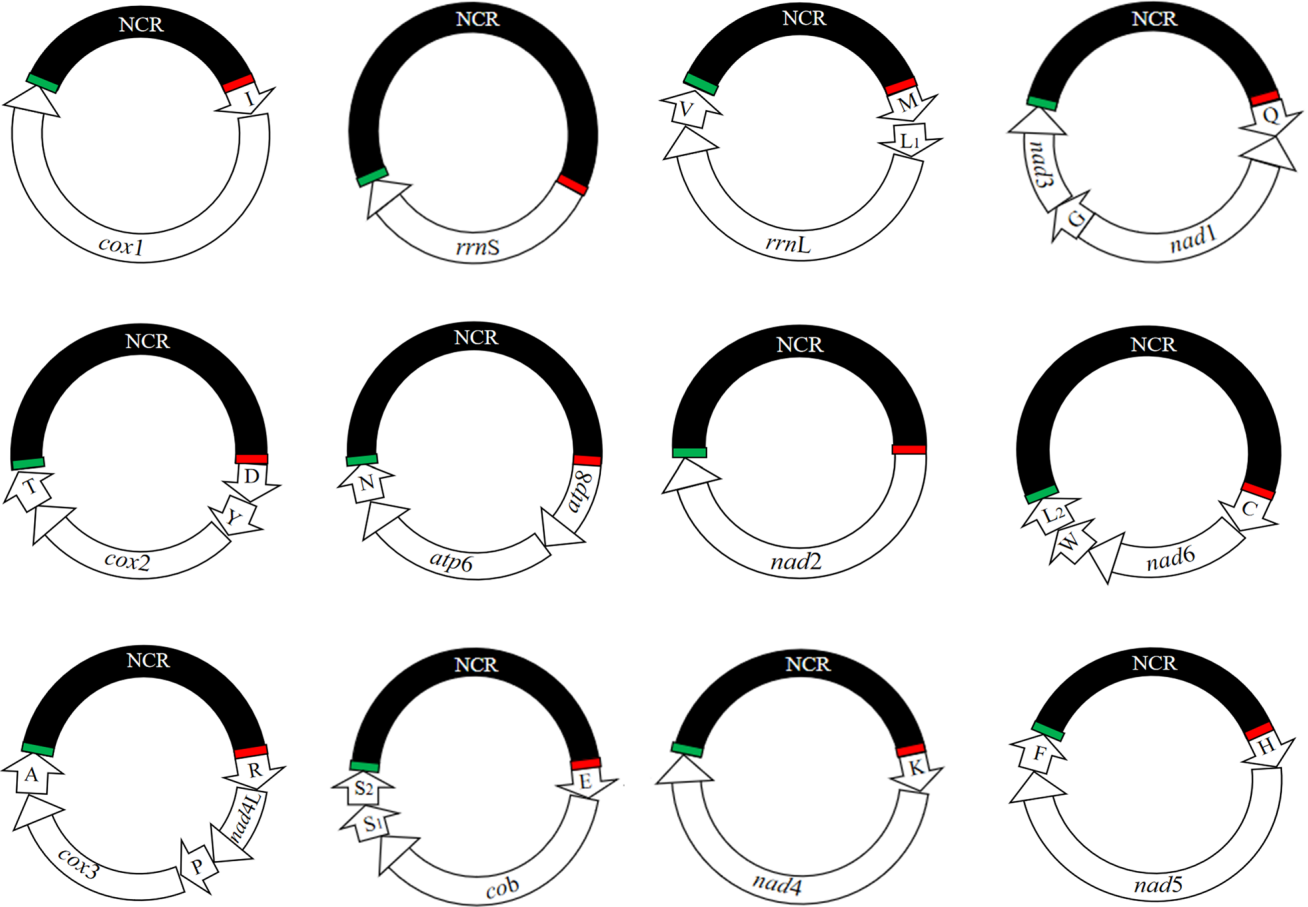
The complete mitochondrial genome of rat louse, *Hoplopleura* sp*.* Each minichromosome has a coding region and a non-coding region (NCR, in black). The names and transcript orientation of genes are indicated in the coding region and the minichromosomes are placed in alphabetical order of protein-coding genes and rRNA genes. *Abbreviations*: *atp*6 and *atp*8, ATP synthase F0 subunits 6 and 8; *co*b, cytochrome b; *cox*1-3, cytochrome *c* oxidase subunits 1–3; *nad*1-6 and *nad*4L, NADH dehydrogenase subunits 1–6 and 4L; *rrn*S and *rrn*L, small and large subunits of ribosomal RNA. tRNA genes are indicated with their single-letter abbreviations of the corresponding amino acids

We sequenced the *Hoplopleura* sp. genome and produced 3 Gb of Illumina short-read sequence data and obtained a total of 6,526,349 × 2 raw reads from adults of *Hoplopleura* sp. After quality filtration, 3,937,826 × 2 clean reads (2 Gb) were generated for assembly of the mt genome. We assembled these sequence-reads into contigs and identified 37 mt genes typical of bilateral animals (Fig. 2; Table 3). These genes are on 12 minichromosomes; each minichromosome is 1.8–2.7 kb in size and consists of a coding region and a non-coding region (NCR) in a circular organization (Table 3). The coding regions have 1–5 genes each and vary in size from 675 to 1760 bp (Table 3). All genes are transcribed in the same direction except for the *nad*1 gene. The nucleotide sequences of the mt minichromosomes of *Hoplopleura* sp. were deposited in the GenBank database under the accession numbers MT792483-MT792494.

**Table 3:** Mitochondrial minichromosomes of the rat louse *Hoplopleura* sp., identified by Illumina sequencing

Minichromosome	Size (bp)	Size of coding region (bp)	Size of non-coding region (bp)	Intergenic region (bp)
I-*cox*1	2531	1549	975	7
*rrn*S	1869	675	1194	0
M-L1-*rrn*L-V	2257	1323	934	0
Q-*nad*1-G-*nad*3	2525	1445	1063	17
D-Y-*cox*2-T	2087	880	1129	78
*atp*8-*atp*6-N	2023	896	1118	9
*nad*2	2141	981	1160	0
C-*nad*6-W-L2	1979	673	1305	1
R-*nad*4L-P-*cox*3-A	2311	1251	1057	3
E-*cyt*b-S1-S2	2417	1304	1113	0
K -*nad*4	2289	1313	975	1
H-*nad*5-F	2695	1759	935	1
Total	27,124	14,049	12,958	117

We sequenced the full-length non-coding regions of all of the 12 mt minichromosomes of the *Hoplopleura* sp., which range from 935 (H-*nad*5-F minichromosome) to 1305 bp (C-*nad*6-W-L_2_ minichromosome) (Table 3). The longest non-coding region of *Hoplopleura* sp. was shorter than the longest non-coding region of other sucking lice known, such as pig lice (2370 bp) [6] and horse lice (3276 bp) [13]. As in the human lice [12], rat lice [7] and pig lice [6], each coding region of *Hoplopleura* sp. is flanked by a conserved non-coding AT-rich motif (88 bp, 71.6%) upstream and a GC-rich motif (39 bp, 79.5%) downstream, indicating functional significance of these motifs in the mt genomes of blood-sucking lice.

### Annotation

The boundaries between protein-coding genes of the mt genome of *Hoplopleura* sp. were determined by aligning its sequence and identifying translation initiation and termination codons with those of *H. kitti* and *H. akanezumi* [7]. *Hoplopleura* sp. mt genome encoded 13 protein-encoding genes, which had four initiation codons (ATT, ATG, TTG and GTG). Among them, both ATT (*nad*2, *nad*4L, *nad*5, *cox*3 and *cyt*b) and ATG (*nad*3, *nad*4, *nad*6, *atp*6 and *atp*8) are the highest frequency of being used as initiation codons. Moreover, TTG (*nad*1 and *cox*2) and GTG (*cox*1) are used in the mt genome. This mt genome has three termination codons (TAA, TAG and T). Among them, TAG is most frequently used (five times altogether), by *cox*1, *nad*2, *nad*3, *nad*4L and *cyt*b. TAA was second in frequency of recurrence (four times) as termination codons, *cox*2, *atp*6, *atp*8 and *nad*4, used it in the mt genome of *Hoplopleura* sp. Furthermore, *cox*3, *nad*1, *nad*5 and *nad*6 genes use T as termination codons. Incomplete terminations (TA and T) of protein-coding genes are commonly found in other mt genomes of blood-sucking lice, including *H. suis* [6], *H. apri* [6], *H. asini* [13], *H. kitti* [7], *P. asiatica* [8], *P. spinulosa* [8], *P. schaeffi* [9], *M. praelongiceps* [11] and *P. pubis* [12]. In the mt genome of *Hoplopleura* sp., the sizes of the *rrn*L and *rrn*S genes were 1125 bp and 675 bp, respectively. The 22 tRNA genes ranged from 59 to 71 bp in size. The secondary structure predictions in *Hoplopleura* sp. (not shown) were similar to those of *H. kitti* and *H. akanezumi* [7].

### Variation in mt minichromosome composition among three rat lice

**Fig. 3:**
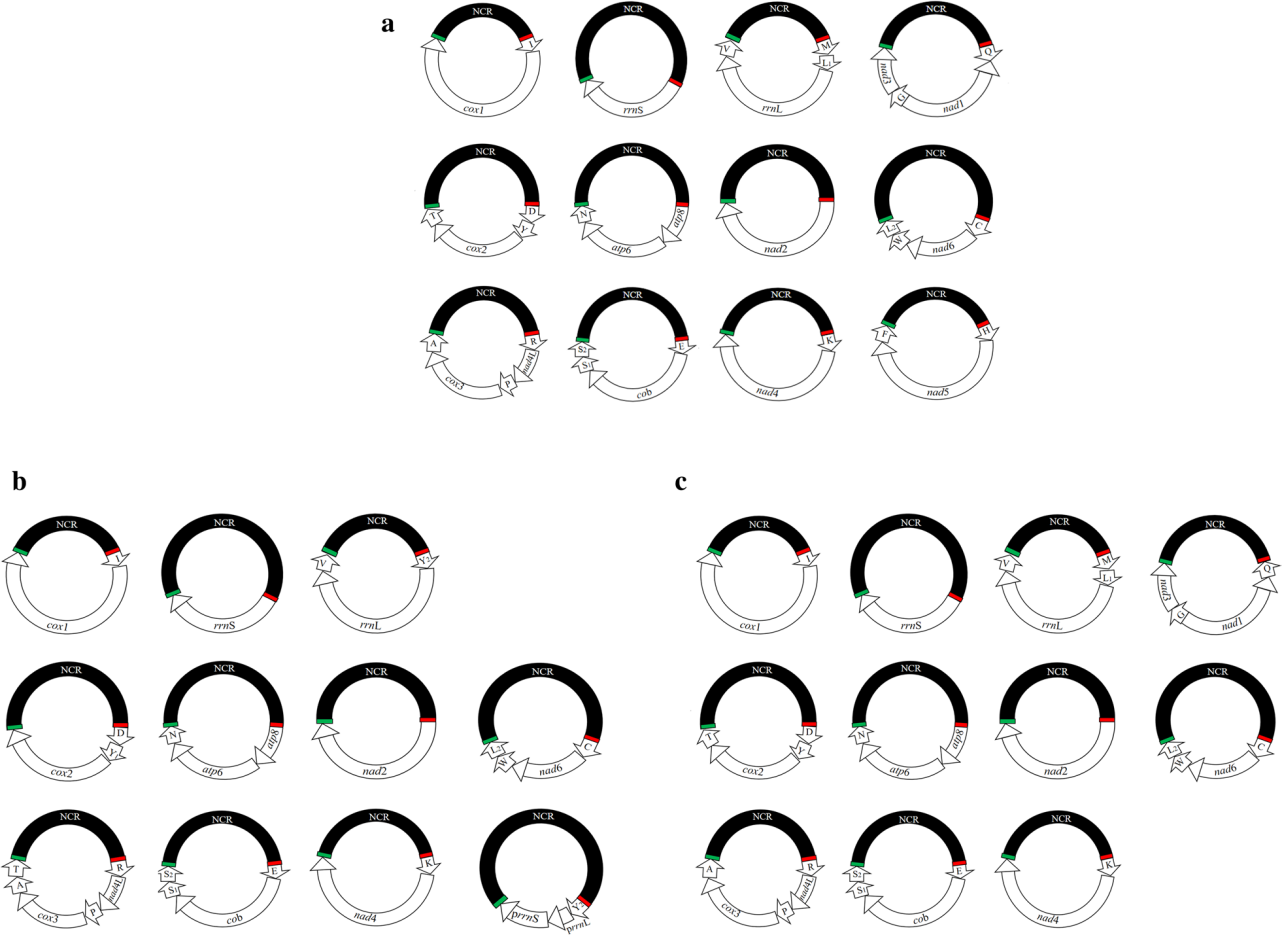
The differences among all minichromosomes of three *Hoplopleura* lice. **a** 12 identified circular minichromosomes of *Hoplopleura* sp. **b** 11 identified circular minichromosomes of *Hoplopleura akanezumi*. **c** 11 identified circular minichromosomes of *Hoplopleura kitti*

The complete mt genome sequences of *Hoplopleura* sp. fragmented into 12 circular minichromosomes. The incomplete mt genomes of *H. kitti* and *H. akanezumi* have 11 identified circular minichromosomes [7]. Eleven minichromosomes of the rat louse, *Hoplopleura* sp., have the same gene content and gene arrangement as their counterparts of the rat louse, *H. kitti*. Eight of these minichromosomes of the rat lice, *Hoplopleura* sp. and *H. kitti*, have the same gene content and gene arrangement as their counterparts of the rat louse, *H. akanezumi* [7]. However, the other two minichromosomes of the rat louse *Hoplopleura* sp. are not present in the rat louse *H. akanezumi* [7]. In *Hoplopleura* sp., one of the minichromosomes has four genes, D-Y-*cox*2-T (Fig. 2); however, in *H. akanezumi* this minichromosome has only three genes, D-Y-*cox*2. Similarly, another minichromosome of *Hoplopleura* sp. has five genes, R-*nad*4L-P-*cox*3-A (Fig. 2); however, in *H. akanezumi* this minichromosome has six genes, R-*nad*4L-P-*cox*3-A-T  (Fig. 3). Interestingly, a chimeric minichromosome has been found in *H. akanezumi* which contains parts of the two rRNA genes, p*rrn*L and p*rrn*S, which are only 5% (51 bp) and 24% (172 bp) of the full-length *rrn*L and *rrn*S, respectively [7]. However, this chimeric minichromosome has not been identified in *H. kitti* and *Hoplopleura* sp.

### Comparative mt genomic analyses of *Hoplopleura *sp. with *H. kitti* and *H. akanezumi*

A comparison of the nucleotide and the amino acid sequences of each protein-encoding gene (except for *nad*1, *nad*3 and *nad*5) of the three *Hoplopleura* species is given in Table 4. Pairwise comparisons of the nucleotide and amino acid sequences revealed identities of 50.6–77.2% and 37.5–90.2% among them, respectively. The greatest nucleotide variation was in the *atp*8 gene (49.4%), whereas the lowest differences (22.8%) were detected in the *cox*1 gene (Table 4). The difference across both concatenated nucleotide and amino acid sequences of the ten protein-coding genes was 37.5% and 36.8% between *Hoplopleura* sp. and *H. kitti*, 36.7% and 34.7% between *Hoplopleura* sp. and *H. akanezumi*, and 34.6% and 33.4% between *H. kitti* and *H. akanezumi*.

**Table 4:** Nucleotide (nt) and/or predicted amino acid (aa) sequence differences in mitochondrial genes among *Hoplopleura* sp. (Hs), *H. kitti* (Hk) and *H. akanezumi* (Ha) upon pairwise comparison

Gene/region	Nt sequence length			Nt difference (%)			Number of aa			aa difference (%)		
	Hs	Hk	Ha	Hs/Hk	Hs/Ha	Hk/Ha	Hs	Hk	Ha	Hs/Hk	Hs/Ha	Hk/Ha
*atp*6	651	651	654	36.34	36.54	33.49	216	216	217	32.26	33.64	27.65
*atp8*	174	195	177	47.50	49.44	46.97	57	64	58	62.50	59.02	54.69
*nad*2	981	990	984	48.44	44.18	43.40	326	329	327	55.15	53.19	54.85
*nad*4	1248	1242	1254	40.38	40.49	41.21	415	413	417	44.84	45.56	44.84
*nad*4L	273	273	270	42.34	39.56	42.12	90	90	89	48.89	44.44	50.00
*nad*6	478	483	474	43.83	44.49	41.74	158	160	157	47.20	50.63	48.75
*cox*1	1485	1530	1530	29.15	29.26	22.80	494	509	509	17.68	15.32	9.80
*cox*2	687	681	684	38.24	34.40	33.77	228	226	227	41.30	32.02	31.00
*cox*3	787	787	789	34.18	34.60	32.57	261	261	262	27.48	29.77	30.92
*cyt*b	1104	1102	1107	34.30	33.97	32.13	367	367	368	29.70	25.27	25.00
*rrn*S	675	737	690	31.80	25.32	33.73						
*rrn*L	1125	1107	1131	28.48	27.55	29.86						

### Phylogenetic relationships

**Fig. 4:**
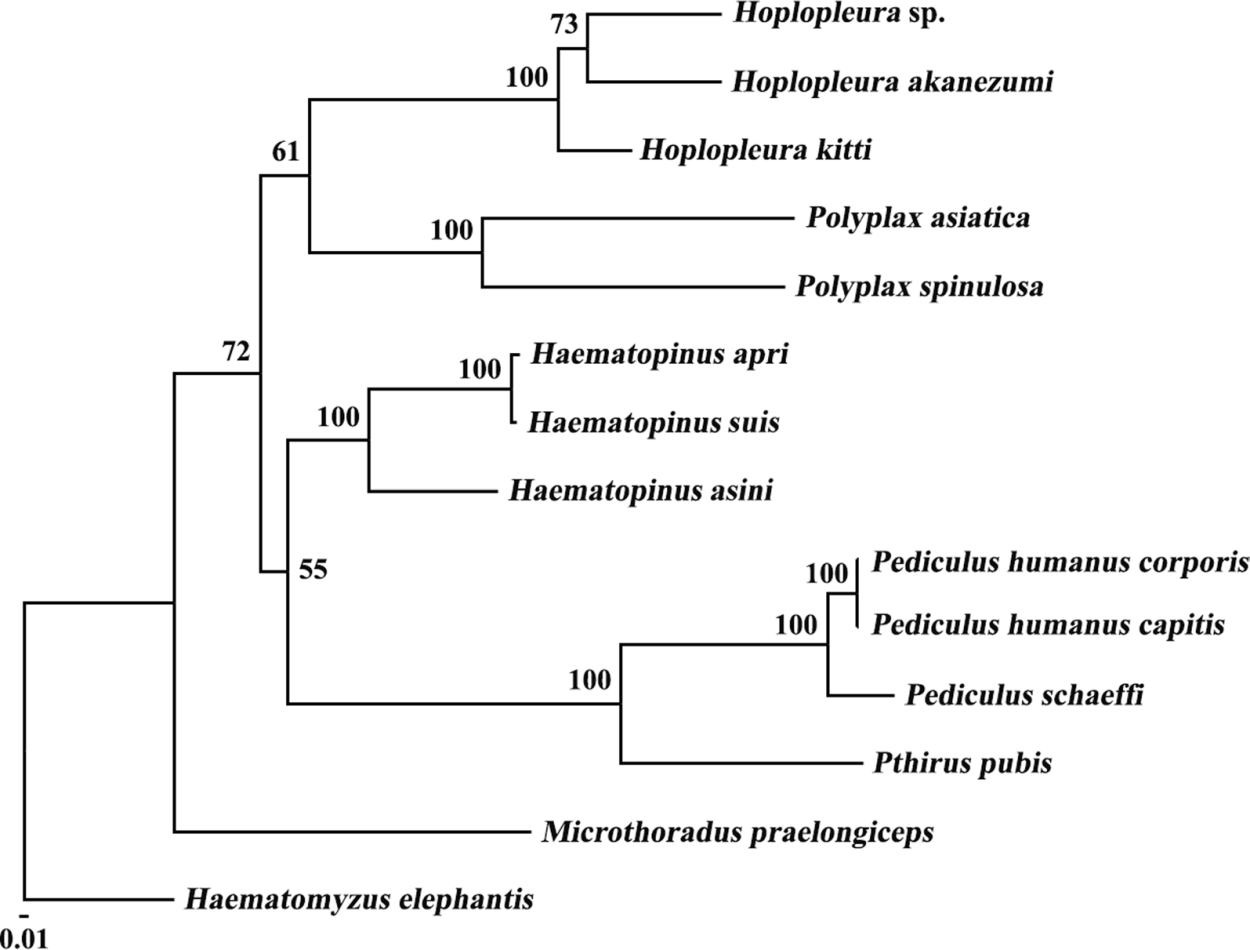
Phylogenetic relationships among 13 species of the suborder Anoplura inferred by maximum likelihood of deduced amino acid sequences of eight mitochondrial proteins using PhyML. The elephant louse, *Haematomyzus elephantis*, was used as the outgroup. Bootstrap values are indicated at nodes

In the present study, phylogenetic analysis of the concatenated amino acid sequence datasets for eight mt protein-coding genes (Fig. 4) showed that the family Hoplopleuridae (*Hoplopleura* sp., *H. kitti* and *H. akanezumi*) clustered to the exclusion of representatives of the families Polyplacidae (*P. asiatica* and *P. spinulosa*), Haematopinidae (*H. apri*, *H. asini* and *H. suis*), Pediculidae (*P. humanus corporis*, *P. humanus capitis* and *P. schaeffi*), Pthiridae (*P. pubis*), and the family Microthoraciidae (*M. praelongiceps*) clustered separately with strong nodal support (bootstrap = 100). Within the family Hoplopleuridae, *Hoplopleura* sp. and *H. akanezumi* clustered together with moderate support (bootstrap value = 73), to the exclusion of *H. kitti*, and then they formed a monophyletic group (bootstrap value = 100). The result was also strongly supported by RAxML analysis (bootstrap value = 100) (Additional file 1: Figure S1).

The work of Johnson et al. [26] created robustness and stability in higher systematics within the order Phthiraptera based on analyses of 1107 single-copy orthologous genes from sequenced genomes of 46 species of lice [26]. Their result has indicated that the genera *Hoplopleura* and *Haematopinus* were more closely related than to the genus *Pediculus* with a strong bootstrap value [26]. However, mt genomic phylogenetic relationships deviated from phylogenies derived from the nuclear genome. Shao et al. [11] performed a phylogenetic analysis with mt genomes, indicating that the genera *Haematopinus* and *Pediculus* were more closely related than to the genus *Hoplopleura* with a strong bootstrap value. Our result also showed the genera *Haematopinus* and *Pediculus* were more closely related than to the genus *Hoplopleura*, but with a weak bootstrap value (bootstrap value = 55) (Fig. 3). Although the number of sucking lice mt genome sequences is increasing, so far, mt genomes of many lineages of sucking lice are underrepresented or not represented. Insufficient taxon sampling for the suborder Anoplura mt genomes might be the cause of the discordance between the mt and nuclear phylogenies.

Many studies have indicated that the mt genome sequence is a valuable genetic marker for phylogenetic studies at various taxonomic levels of different organisms [27, 28], including lice [14, 15]. The fragmentation of the mt genome may have arisen independently in multiple louse clades. Therefore, the mt genome sequences of rat louse *Hoplopleura* sp. could promote to reassess the systematic relationships of lice within the suborder Anoplura using mt genomic datasets. No species from the other genera (*Ancistroplax*, *Ferrisella*, *Haematopinoides*, *Paradoxophthirus*, *Pterophthirus*, *Schizophthirus* and *Typhlomyophthirus*) within the family Hoplopleuridae was included in our analyses. Therefore, more expanded taxa sampling is necessary for future phylogenetic studies of the family Hoplopleuridae using mt genomic datasets.

## Conclusions

Comparison among the three rat lice revealed variation in the composition of mt minichromosomes among species of the genus *Hoplopleura*. *Hoplopleura* sp. is the first species from the family Hoplopleuridae for which a complete fragmented mt genome has been sequenced. The new data provide useful genetic markers for studying the population genetics, molecular systematics and phylogenetics of blood-sucking lice.

## Supplementary information

**Additional file 1: Figure S1.** Phylogenetic relationships among 13 species of the suborder Anoplura inferred from maximum likelihood of deduced amino acid sequences of 8 mitochondrial proteins using RAxML. One elephant louse, *Haematomyzus elephantis* was used as the outgroup. Bootstrap values were indicated at nodes.

## Data Availability

The fragmented mitochondrial genome sequences of *Hoplopleura* sp. from the Edward’s long-tailed rats have been deposited in the GenBank database under the accession numbers MT792483–MT792494.

## Abbreviations

mt: mitochondrial
rDNA: ribosomal DNA
*nad*1: NADH dehydrogenase subunit 1
*nad*3: NADH dehydrogenase subunit 3
*nad*5: NADH dehydrogenase subunit 5
*cox*1: cytochrome *c* oxidase subunit 1
*rrn*S: small subunit of rRNA
*rrn*L: large subunit of rRNA
tRNA: transfer RNA
*nad*2: NADH dehydrogenase subunit 2
*nad*4: NADH dehydrogenase subunit 4
*nad*4L: NADH dehydrogenase subunit 4L
*cox*3: cytochrome *c* oxidase subunit 3
*cyt*b: cytochrome b
*nad*6: NADH dehydrogenase subunit 6
*atp*6: ATP synthase F0 subunit 6
*atp*8: ATP synthase F0 subunit 8
*cox*2: cytochrome *c* oxidase subunit 2
