# Supplementary figures and images for: Variation of mitochondrial minichromosome composition in Hoplopleura lice (Phthiraptera: Hoplopleuridae) from rats
Source: Parasit Vectors. 2020 Oct 6;13:506. doi: 10.1186/s13071-020-04381-y (PMC7539455; doi:10.1186/s13071-020-04381-y)

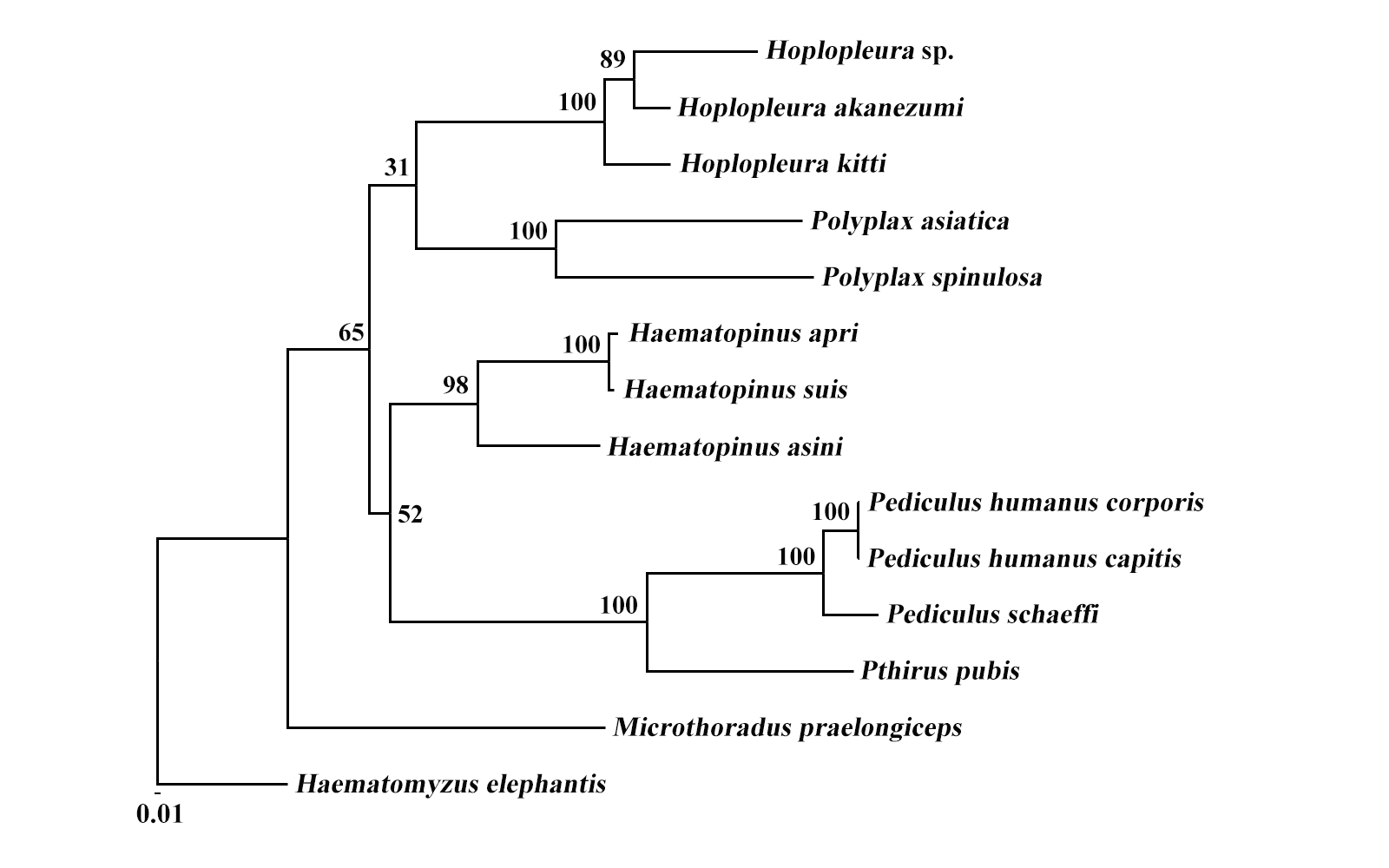

Supplement: Supplementary file 1 — Additional file 1: Figure S1. Phylogenetic relationships among 13 species of the suborder Anoplura inferred from maximum likelihood of deduced amino acid sequences of 8 mitochondrial proteins using RAxML. One elephant louse, Haematomyzus elephantis was used as the outgroup. Bootstrap values were indicated at nodes. [file 13071_2020_4381_MOESM1_ESM.tif]
